# Butyrylated Starch Attenuates Antibiotic‐Exacerbated Allergic Rhinitis by Restoring the Gut Microbiota and Barrier Function in Mice

**DOI:** 10.1002/fsn3.72000

**Published:** 2026-06-07

**Authors:** Yuxi Lin, Qingqing Xu, Siyi Tang, Jingmin Chen, Xing Lin, Yang Liu, Yuanteng Xu

**Affiliations:** ^1^ Department of Otorhinolaryngology‐Head and Neck Surgery The First Affiliated Hospital of Fujian Medical University Fuzhou China; ^2^ Department of Otorhinolaryngology‐Head and Neck Surgery, National Regional Medical Center Binhai Campus of The First Affiliated Hospital of Fujian Medical University Fuzhou China; ^3^ Department of Otorhinolaryngology Fujian Children's Hospital Fuzhou China; ^4^ Department of Otorhinolaryngology‐Head and Neck Surgery West China Second University Hospital, Sichuan University Sichuan China; ^5^ Allergy Center The First Affiliated Hospital of Fujian Medical University Fuzhou China

**Keywords:** allergic rhinitis, butyrylated starch, gut microbiota, intestinal barrier function, regulatory T cells, vancomycin

## Abstract

Allergic rhinitis (AR) is often exacerbated by antibiotic‐induced gut dysbiosis. This study investigated whether butyrylated starch (BS) could mitigate vancomycin (VAN)‐induced AR aggravation by restoring gut homeostasis. In a murine AR model (*n* = 5 per group), VAN administration significantly depleted the abundance of butyrate‐producing bacteria (e.g., *Clostridia* and *Lachnospiraceae*), suppressed colonic butyrate levels (1.08 ± 0.36 vs. 8.20 ± 0.78 μg/g in controls), and impaired intestinal barrier integrity, thereby worsening nasal symptoms (sneezing frequency: 4.40 ± 1.14 vs. 2.40 ± 0.55 in the AR group). Intervention with BS, a targeted butyrate precursor, effectively reversed these changes by increasing the abundance of butyrogenic taxa, increasing butyrate production (22.86 ± 3.27 μg/g), upregulating the expression of tight junction proteins (Occludin‐1, ZO‐1, and Claudin‐3), and increasing the number of splenic regulatory T (Treg) cells. Our findings demonstrate that BS ameliorates antibiotic‐induced AR via coordinated restoration of the gut microbiota‐barrier‐immune axis, suggesting a novel dietary strategy for the management of allergic inflammation.

## Graphical abstract

Vancomycin‐induced gut dysbiosis depletes butyrate‐producing bacteria, reduces colonic butyrate, disrupts the intestinal barrier, and exacerbates allergic rhinitis (AR). The colon‐targeted butyrate precursor butyrylated starch reverses this pathological cascade, restores immune homeostasis, and alleviates AR, providing a novel dietary intervention for antibiotic‐exacerbated AR.

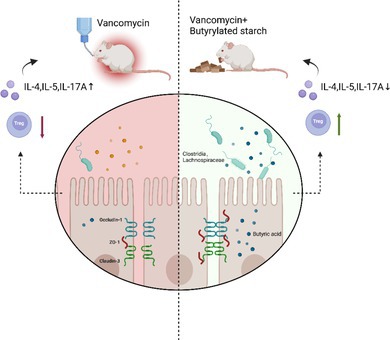

## 1 Introduction

Allergic rhinitis (AR) is a highly prevalent chronic inflammatory disorder of the nasal mucosa characterized by aberrant Type 2 and Type 17 immune responses to innocuous environmental allergens. With a global prevalence approaching 30%, AR represents a major public health burden worldwide: AR not only causes debilitating nasal symptoms, including congestion, pruritus, and rhinorrhea, but also significantly impairs patients' quality of life, sleep efficiency, work productivity, and cognitive function (Pawankar et al. 2012). Current first‐line treatments for AR, including antihistamines and intranasal corticosteroids, provide only symptomatic relief and fail to address the underlying immunological and microbial drivers of disease progression and exacerbation (Wheatley and Togias 2015). Thus, there is an urgent unmet need for safe, mechanism‐based interventions that target the root causes of AR.

The widespread inappropriate use of antibiotics in clinical practice is a well‐established driver of persistent gut microbiota dysbiosis (Becattini et al. 2016; Fujisaka et al. 2016), and accumulating evidence from epidemiological and preclinical studies has linked antibiotic exposure to an increased risk of developing and exacerbating allergic diseases, including AR (Zanvit et al. 2015; Yang et al. 2014; Kim et al. 2018; Lee et al. 2020; Ni et al. 2019). Our previous preclinical work demonstrated that the use of vancomycin, a commonly used glycopeptide antibiotic, markedly exacerbated nasal inflammation in a murine AR model through the induction of profound gut dysbiosis and disruption of the production of short‐chain fatty acids (SCFAs), the key immunomodulatory metabolites of the gut microbiota (Chen et al. 2022). A homeostatic gut microbiota is critical for maintaining intestinal barrier integrity, primarily by promoting the expression of epithelial tight junction proteins, including zonula occludens‐1 (ZO‐1) and Claudin‐3. Disruption of this physical barrier increases intestinal permeability (a state known as the “leaky gut”), enabling the translocation of proinflammatory molecules such as lipopolysaccharide (LPS) into the systemic circulation, which drives systemic low‐grade inflammation and amplifies pathological immune responses at distal mucosal sites, including the nasal mucosa (Régnier et al. 2021; Desai et al. 2016; Stevens et al. 2018).

Butyrate, the dominant SCFA produced by the anaerobic fermentation of dietary fiber in the colon, is a central mediator of the crosstalk between the gut microbiota and host immunity. It exerts well‐documented protective effects on intestinal barrier integrity and is a potent epigenetic and immunomodulatory regulator that maintains systemic immune homeostasis (Blaak et al. 2020; Morrison and Preston 2016). Exogenous sodium butyrate supplementation has shown anti‐inflammatory efficacy in multiple preclinical models of inflammatory disease through the modulation of immune cell function and the repair of intestinal barrier damage (Hu et al. 2018). Similarly, butyrate precursors such as tributyrin have been shown to alleviate antibiotic‐induced gut dysbiosis and mucosal injury (Yang et al. 2023). However, these conventional butyrate formulations have critical translational limitations: they are rapidly absorbed and metabolized in the upper gastrointestinal tract, with minimal amounts reaching the colon, the primary site of the physiological activity of butyrate (Zhang et al. 2025).

To overcome this limitation, colon‐targeted butyrate delivery systems have been developed, among which butyrylated starch (BS) is a promising food‐grade candidate. BS is a starch‐based butyrate precursor that resists hydrolysis by upper gastrointestinal amylases and is specifically fermented by the colonic microbiota to release butyrate locally (Zhang et al. 2025, 2024). Our previous work provided the first evidence that BS supplementation ameliorates AR symptoms in mice (Chen et al. 2024), but its therapeutic potential and underlying mechanisms in the context of antibiotic‐exacerbated AR remain completely uncharacterized. While antibiotics are known to profoundly deplete butyrate‐producing commensal bacteria, including *Clostridia* and *Lachnospiraceae* (Hays et al. 2024), and to drive persistent intestinal barrier dysfunction and heightened allergic susceptibility (Zhang et al. 2021; Geirnaert et al. 2017), no studies to date have investigated whether BS can reverse antibiotic‐induced AR exacerbation by targeting the gut microbiota‐barrier‐immune axis.

On the basis of this rationale, we hypothesized that BS supplementation mitigates vancomycin‐induced AR aggravation in mice by restoring the abundance of butyrate‐producing gut bacteria, increasing colonic butyrate levels, repairing intestinal barrier integrity, and promoting regulatory T (Treg) cell‐mediated immune homeostasis. This study aimed to systematically characterize the therapeutic efficacy of BS against antibiotic‐exacerbated AR and elucidate the underlying mechanisms involving the coordinated regulation of the gut microbiota‐barrier‐immune axis.

## 2 Materials and Methods

### 2.1 Animals

BALB/c mice (3 weeks old, weighing 14–17 g) were purchased from Shanghai SLAC Laboratory Animal Co. Ltd. (Production License No. SCXK [Hu] 2022‐0004). All the mice were housed under specific pathogen‐free (SPF) conditions at the Experimental Animal Center of Fujian Medical University in a standardized environment (12/12 h light/dark cycle, 22°C ± 2°C, 55% ± 10% humidity) with free access to autoclaved water and standard rodent chow. The bedding was changed every 3 days. After a 1‐week acclimatization period, all the experimental procedures commenced. The study protocol was approved by the Institutional Animal Care and Use Committee (IACUC) of Fujian Medical University (Approval No. FJMU 2023‐0170), and all the procedures were performed in accordance with the relevant guidelines and regulations.

### 2.2 Preparation and Characterization of Butyrylated Starch

On the basis of previous research (Li et al. 2021), the butyrylated starch (BS) used in this study was kindly provided by the research group of Prof. Hong from the School of Food Science and Technology, Jiangnan University (Wuxi, China).

The degree of substitution (DS), which indicates the average number of hydroxyl groups substituted by butyryl moieties per glucose unit, was determined according to the analytical principle outlined in the Chinese National Standard GB 29923‐2013 (“Determination Method of Acetyl Group Content in Acetate Starch”). This standard method involves alkaline hydrolysis of the ester bonds and titration of the liberated acid. The DS value for the prepared BS sample was calculated as 0.237 according to the standard calculation procedure. More details in this regard are available in the Supporting Informations and Table S1.

### 2.3 Induction and Grouping of the AR Animal Model

**FIGURE 1:**
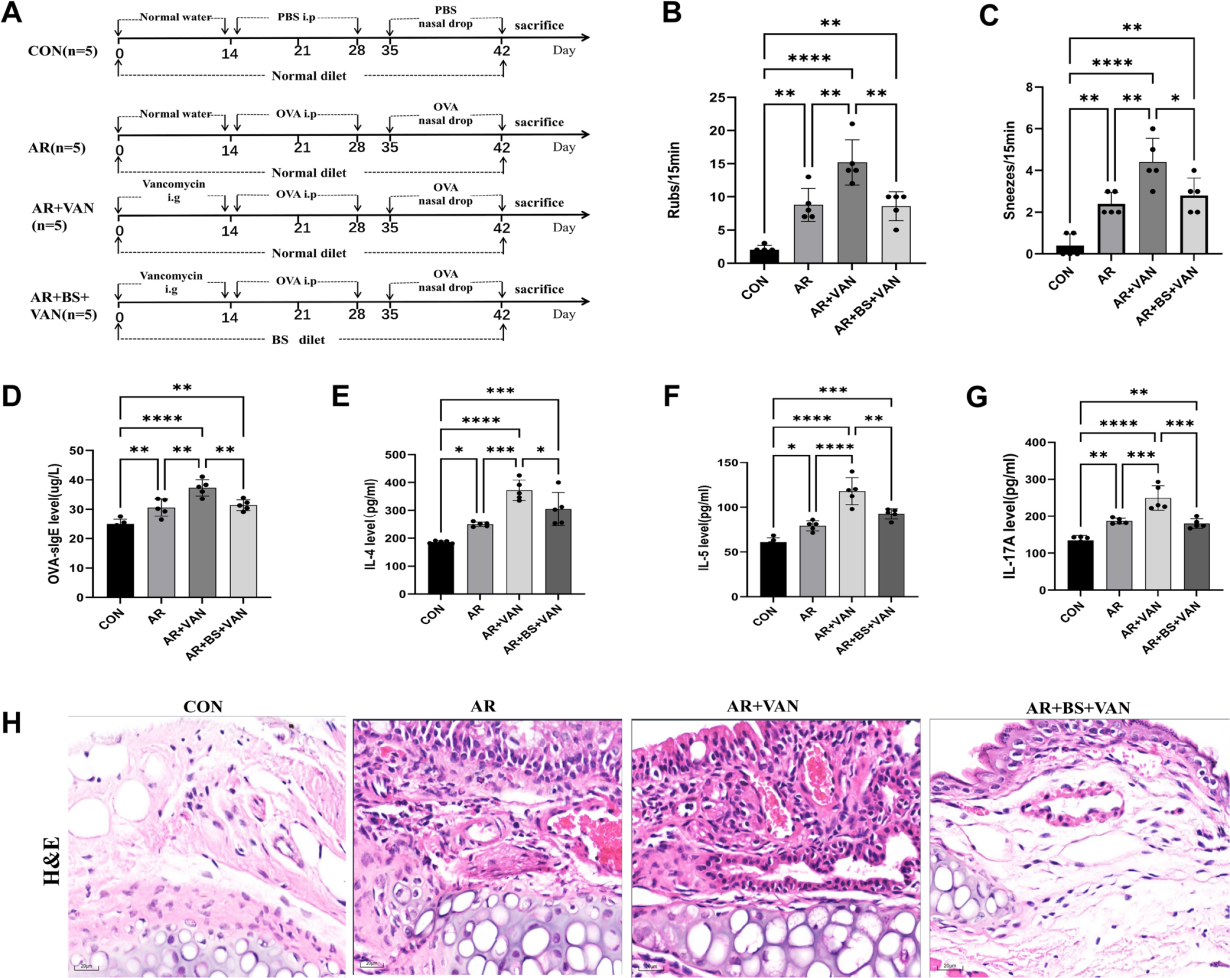
Butyrylated starch alleviates vancomycin‐induced allergic rhinitis symptoms and systemic inflammation. (A) Experimental timeline. i.p., intraperitoneal; i.g., intragastric. (B, C) Quantification of nasal scratching (B) and sneezing (C) events within 15 min after the last challenge. (D–G) Serum levels of OVA‐sIgE (D), IL‐4 (E), IL‐5 (F), and IL‐17A (G) as determined by ELISA. (H) Representative hematoxylin and eosin (H&E)‐stained sections of nasal mucosa. Scale bar, 100 μm. The data are presented as the means ± SDs (*n* = 5). *p < 0.05, **p < 0.01, ***p < 0.001, ****p < 0.0001 (one‐way ANOVA with Tukey's post hoc test).

Twenty BALB/c mice were randomly divided into four groups (5 mice/group): the control group (Con), AR model group (AR), vancomycin‐treated group (VAN + AR), and butyrylated starch‐fed plus vancomycin‐treated group (BS + VAN + AR), referring to Figure 1A. From Day 0 to Day 14, 10 μL of vancomycin hydrochloride (100 mg/kg/day, dissolved in sterile water) was administered daily via oral gavage per gram of body weight. In the butyrylated starch group, butyrylated starch was introduced simultaneously. On Days 14, 21, and 28, the mice were sensitized via intraperitoneal injection with a mixture of 40 μg of ovalbumin (Sigma‐Aldrich, A5503) adsorbed in 100 μL of alum adjuvant (Imject Alum, Thermo Fisher) and 100 μL of phosphate‐buffered saline (PBS). From Days 35 to 41, nasal challenge with OVA (40 mg/mL) was performed. Fifteen minutes after the last challenge, behavioral symptoms were observed. On Day 42, the mice were anesthetized, and blood and tissue samples from the orbital blood, nasal mucosa, colonic tissue, colonic feces, and spleen lymph nodes were collected.

### 2.4 Evaluation of Nasal Symptoms

Five minutes after the final nasal challenge, the mice were placed in observation cages. A blinded observer unaware of the groupings recorded the number of sneezes and nose scratching episodes for each group over a 15‐min period. Cumulative symptom scores were calculated by summing the graded scores of sneezing and nasal scratching (grading criteria: Table S2), where higher scores indicate more severe AR symptoms.

### 2.5 Serum Cytokine Detection

On Day 42, orbital blood samples were collected from the mice, after which the serum was separated by centrifugation. Serum levels of OVA‐specific IgE (OVA‐sIgE), interleukin (IL)‐4, IL‐5, IL‐17A, and lipopolysaccharide (LPS) were quantified via commercial enzyme‐linked immunosorbent assay (ELISA) kits (OVA‐sIgE: MSK, 69‐210251; IL‐4: MSK, 59‐20069; IL‐5: Boster, EK0408; IL‐17A: MultiSciences, EK217/2‐96; LPS: MSK, KT37561) according to the manufacturers' instructions.

### 2.6 Histopathological Evaluation of Nasal Tissue

Nasal mucosa and colonic tissues were fixed in formalin for 72 h, dehydrated, and embedded in paraffin. Coronal sections (3 μm) were prepared and stained with hematoxylin and eosin (H&E). Pathological changes in the stained sections were observed under an optical microscope and imaged via an imaging system.

### 2.7 Flow Cytometry Analysis

On Day 42, the spleen tissues were harvested and mechanically dissociated into single‐cell suspensions via a sterile 70‐μm cell strainer to remove tissue debris. The cells were subsequently washed twice with phosphate‐buffered saline (PBS) supplemented with 2% fetal bovine serum (FBS) and resuspended at a concentration of 1 × 10^6^ cells/mL. For surface staining, the cells were incubated with fluorescently labeled anti‐CD4 and anti‐CD25 monoclonal antibodies in the dark at 4°C for 30 min. After surface staining, the cells were fixed and permeabilized with commercial Foxp3/Transcription Factor Staining Buffer according to the manufacturer's protocol. For intracellular staining, fixed, permeabilized cells were incubated with a fluorescently labeled anti‐FOXP3 monoclonal antibody in the dark at 4°C for 45 min. Isotype‐matched control antibodies were used to set gating thresholds and exclude nonspecific staining.

Flow cytometric analysis was performed via a commercial flow cytometer, and the data were analyzed via flow cytometry analysis software. The gating strategy was as follows: (1) Gating on single cells by forward scatter area (FSC‐A) versus forward scatter height (FSC‐H) to exclude cell aggregates; (2) excluding dead cells via a fixable viability dye; (3) identifying CD4^+^ T cells by gating on CD4‐positive populations in the CD4 versus side scatter (SSC‐A) dot plot; (4) further gating on CD4^+^CD25^+^ cells within the CD4^+^ T‐cell subset; and (5) defining CD4^+^CD25^+^FOXP3^+^ regulatory T (Treg) cells by selecting FOXP3‐positive cells within the CD4^+^CD25^+^ population.

### 2.8 Western Blot Analysis

Colon tissue proteins were extracted via RIPA lysis buffer supplemented with protease inhibitors. Protein concentrations were determined via a BCA assay kit (Beyotime, P0012). Equal amounts of protein (20–30 μg per lane) were separated via 10% SDS‐PAGE and transferred onto PVDF membranes. The membranes were blocked with 5% nonfat milk for 1 h at room temperature and then incubated overnight at 4°C with primary antibodies against ZO‐1 (Abcam, ab96587), Occludin‐1 (Abcam, ab216327), Claudin‐3 (Invitrogen, 34‐1700), and β‐actin (Immunoway, YM3028) as loading controls. After being washed, the membranes were incubated with appropriate HRP‐conjugated secondary antibodies for 1 h at room temperature. The protein bands were visualized via an enhanced chemiluminescence (ECL) substrate (Millipore) and quantified via densitometry using Image‐Pro Plus software (version 6.0).

### 2.9 PCR Amplification and 16S rDNA Sequencing

Microbial genomic DNA was extracted from frozen fecal samples via the CTAB/SDS method. The hypervariable V3–V4 region of the 16S rRNA gene was amplified with barcoded primers. The PCR products were subsequently purified and quantified, and sequencing libraries were constructed via the NEBNext Ultra DNA Library Prep Kit (NEB). 16S rRNA gene sequencing was performed on the Illumina NovaSeq platform, generating paired‐end (PE) raw reads. Raw Fastq files were processed following a standardized bioinformatics pipeline consistent with classic 16S data analysis workflows to ensure reproducibility and data quality, with detailed software versions and parameter settings as follows: Raw sequences were first demultiplexed by unique barcode sequences to eliminate cross‐sample contamination. Subsequent quality control and sequence assembly were conducted via Pear software (v0.9.11). Sequences containing ambiguous bases (N) or primer mismatches were discarded, bases with a quality score < Q20 were trimmed, and paired‐end reads were merged with a minimum overlap length of 10 bp and a *p* value threshold of 0.0001 to guarantee reliable assembly. Chimeric sequences and low‐quality short fragments were removed via Vsearch (v2.27.0) and qc_fasta_v2 (v2.0) software: chimeric sequences were identified and filtered via the UCHIME algorithm, with known chimeras excluded by alignment against a reference database and de novo chimeras eliminated through self‐alignment of assembled sequences; noncompliant short sequences were discarded simultaneously. Finally, the clean sequences were clustered into operational taxonomic units (OTUs) at a 97% sequence similarity threshold via Vsearch (v2.27.0), and taxonomic annotation was performed against the SILVA SSU r138 database with a confidence threshold of 0.8 to assign taxonomic ranks (phylum, class, order, family, genus).

### 2.10 Short‐Chain Fatty Acid (SCFA) Analysis by GC‐QqQ‐MS‐Targeted Metabolomics

Intestinal content samples from 20 mice (four groups: Con, AR, Van, BS_Van; *n* = 5 per group) were subjected to targeted metabolomic analysis to quantify SCFA levels via gas chromatography‐triple quadrupole mass spectrometry (GC‐QqQ‐MS). Raw paired‐end (PE) data in Fastq format were processed for quality control and preprocessing to ensure data reliability. Briefly, missing values were filtered by retaining peak area data with no more than 50% missing values in a single group or across all groups; the remaining missing values were imputed by multiplying the minimum value of the corresponding metabolite by a random number between 0 and 0.5. A total of 11 SCFAs were retained after preprocessing, and the processed peak area data were exported as specified. The quality control (QC) samples (pooled aliquots of all the experimental samples) were analyzed alongside the experimental samples to assess method reproducibility, with the relative standard deviation (RSD) of the peak areas for all the SCFAs in the QC samples < 15%.

### 2.11 Statistics

The data are presented as the means ± standard deviations (SDs). Normality was assessed via the Shapiro–Wilk test, and homogeneity of variance was confirmed via Levene's test. Comparisons between two groups were performed via an unpaired two‐tailed Student's *t*‐test. For comparisons among multiple groups, one‐way analysis of variance (ANOVA) was employed, followed by Tukey's post hoc test for multiple comparisons. For data that did not meet the assumptions of normality or equal variance, the nonparametric Kruskal–Wallis test with Dunn's post hoc test was used. A *p* value < 0.05 was considered to indicate statistical significance. All the statistical analyzes were performed via SPSS (version 26.0), and the graphs were generated via GraphPad Prism (version 9.5).

### 2.12 Correlation Analysis

Spearman rank‐order correlation analysis was performed to assess the monotonic relationships between butyric acid levels, key butyrate‐producing bacterial taxa (at the class, order, family, and genus levels), and indicators of systemic inflammation (OVA‐sIgE, IL‐4, IL‐5, and IL‐17A) across all individual mice (*n* = 20). Correlation coefficients (*r*) and *p* values were calculated. A correlation matrix heatmap was generated to visualize the network of associations, and significant bivariate correlations (*p* < 0.05) are presented as scatter plots. These analyzes were performed via R4.3.3.

## 3 Results

### 3.1 Butyrylated Starch Alleviates Vancomycin‐Exacerbated Allergic Symptoms in Mice

To investigate the therapeutic potential of butyrylated starch (BS) for antibiotic‐induced allergic rhinitis (AR), we established four experimental groups: control (Con), AR, AR with vancomycin (AR + VAN), and AR + VAN with BS supplementation (AR + BS + VAN) (Figure 1A). Compared with the Con group, the AR group presented a significantly greater frequency of sneezing and nasal scratching events (*p* < 0.01). Specifically, the sneezing frequency (events/15 min) increased from 0.40 ± 0.55 (Con) to 2.40 ± 0.55 (AR). Compared with AR treatment, vancomycin treatment markedly exacerbated these AR symptoms, with the AR + VAN group showing significantly higher sneezing counts (4.40 ± 1.14 vs. 2.40 ± 0.55, *p* < 0.01). Importantly, supplementation with BS substantially attenuated this exacerbation, as evidenced by significantly lower sneezing frequencies in the AR + BS + VAN group than in the AR + VAN group (2.80 ± 0.84 vs. 4.40 ± 1.14, *p* < 0.01) (Figure 1B,C). Consistent with the individual symptom counts, the cumulative symptom score was significantly lower in the AR + BS + VAN group than in the AR + VAN group (*p* < 0.0001) and was comparable to that in the AR group. These findings confirm that BS supplementation effectively mitigates the overall severity of vancomycin‐exacerbated AR symptoms (Figure S1).

### 3.2 Butyrylated Starch Attenuates Systemic Inflammation and Nasal Mucosa Pathology

Consistent with the behavioral symptoms, the serum levels of OVA‐sIgE and the cytokines IL‐4, IL‐5, and IL‐17A were significantly elevated in the AR group compared with those in the Con group (*p* < 0.05). These proinflammatory biomarkers were further increased in the AR + VAN group (*p* < 0.05 vs. the AR group). However, BS intervention significantly reduced the levels of OVA‐sIgE, IL‐4, IL‐5, and IL‐17A in the AR + BS + VAN group compared with those in the AR + VAN group (*p* < 0.05) (Figure 1D–G). Histopathological examination of the nasal mucosa revealed an intact tissue structure in the Con group. In contrast, the AR group exhibited evident inflammatory damage, including inflammatory cell infiltration, capillary dilation, and glandular hyperplasia. These pathological changes were more severe in the AR + VAN group. Notably, BS supplementation attenuated nasal inflammation and tissue damage (Figure 1H).

### 3.3 Butyrylated Starch Specifically Elevates Colonic Butyrate Levels

**FIGURE 2:**
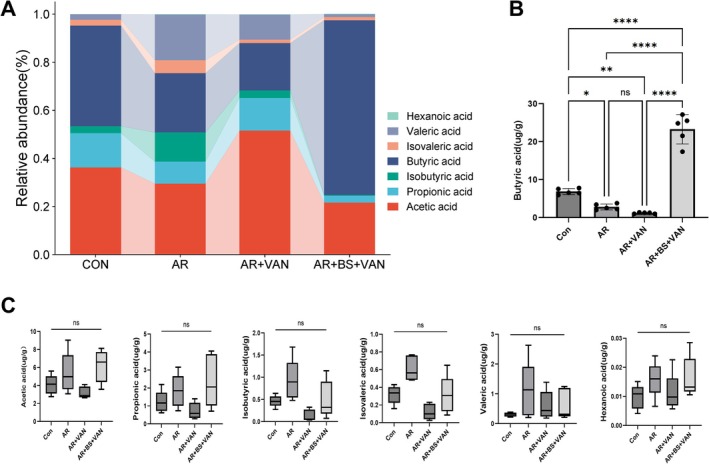
Butyrylated starch specifically increases colonic butyrate levels. (A) Relative abundance (%) of short‐chain fatty acids (SCFAs) in the colonic contents. (B, C) Absolute concentrations (μg/g) of butyrate (B) and other SCFAs (acetate, propionate, valerate, etc.) (C) in the colonic contents. The data are presented as the means ± SDs (*n* = 5). ns, not significant; *p < 0.05, ****p < 0.0001 (one‐way ANOVA with Tukey's post hoc test).

We next assessed whether the protective effects of BS were associated with changes in short‐chain fatty acid (SCFA) production. Targeted metabolomic analysis of the colonic contents revealed a significant reduction in butyrate levels in the AR group (5.76 ± 2.54 μg/g) compared with those in the Con group (8.20 ± 0.78 μg/g, *p* < 0.05). Butyrate levels further decreased in the AR + VAN group (1.08 ± 0.36 μg/g). Crucially, BS intervention dramatically increased the concentration of butyrate in the AR + BS + VAN group (22.86 ± 3.27 μg/g) compared with that in the AR + VAN group (1.08 ± 0.36 μg/g, *p* < 0.0001) (Figure 2A,B). For the other SCFAs, including acetate and propionate, no statistically significant differences were observed among the four groups (Figure 2C). Notably, the levels of acetate and propionate, two SCFAs that are metabolically associated with butyrate, tended to decrease distinctly in the AR + VAN group compared with those in the AR group; after BS intervention, the colonic levels of these SCFAs in the AR + BS + VAN group recovered to a certain degree. However, neither the decrease in the AR + VAN group nor the recovery in the AR + BS + VAN group reached statistical significance. This result may be explained by the fact that, compared with butyrate, butyrylated starch is designed as a targeted butyrate precursor: its core biological function focuses on enriching butyrate‐producing microbiota and promoting butyrate synthesis, leading to a much weaker regulatory effect on acetate and propionate.

### 3.4 Butyrylated Starch Restores Intestinal Barrier Integrity

**FIGURE 3:**
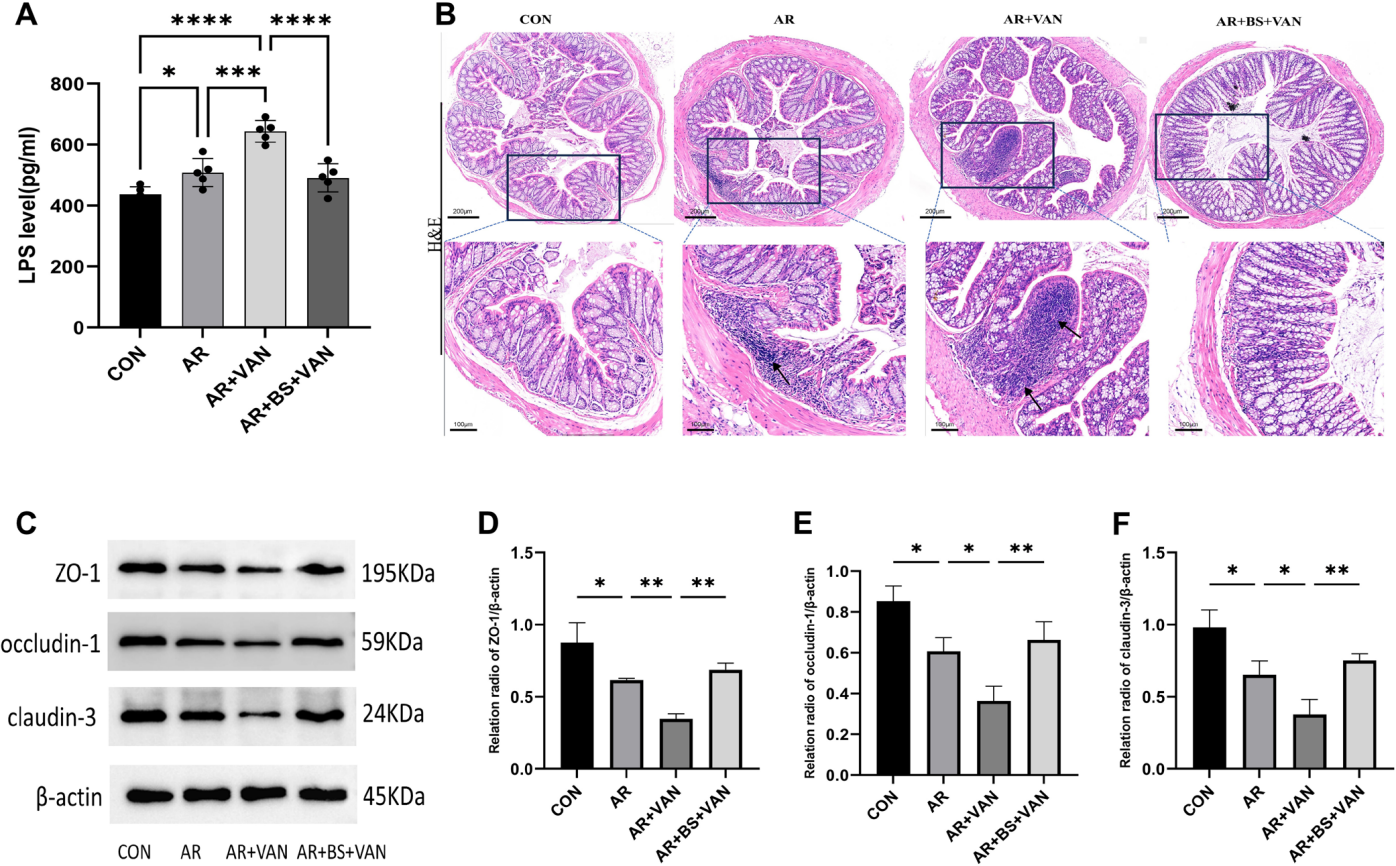
Butyrylated starch restores intestinal barrier integrity. (A) Serum lipopolysaccharide (LPS) levels. (B) Representative H&E‐stained sections of colon tissue. The black arrows indicate abnormal pathological areas, including inflammatory cell infiltration and crypt architecture disruption. Scale bars: 200 μm (Upper panels) and 100 μm (lower panels). (C) Representative western blot bands showing the expression of ZO‐1, Occludin‐1, and Claudin‐3. β‐Actin served as the loading control. (D–F) Densitometric quantification of ZO‐1 (D), Occludin‐1 (E), and Claudin‐3 (F) protein levels normalized to those of β‐Actin. The data are presented as the means ± SDs (*n* = 3–6). *p < 0.05, **p < 0.01, ***p < 0.001, ****p < 0.0001 (one‐way ANOVA with Tukey's post hoc test).

Given the critical role of butyrate in maintaining gut barrier function, we evaluated intestinal integrity. Serum levels of lipopolysaccharide (LPS), a marker of gut barrier leakage, were significantly elevated in the AR group but drastically increased in the AR + VAN group (*p* < 0.0001 vs. AR). BS supplementation significantly reduced the serum LPS concentration (*p* < 0.0001) (Figure 3A). H&E staining of colonic tissues revealed inflammatory changes, including goblet cell loss, crypt architecture disruption, and immune cell infiltration, in the AR group, which were severely exacerbated by vancomycin. BS treatment markedly alleviated these pathological features (Figure 3B). At the molecular level, the expression of key tight junction proteins (Occludin‐1, ZO‐1, and Claudin‐3) was significantly downregulated in the AR and AR + VAN groups. BS intervention effectively upregulated the expression of these genes (*p* < 0.01 vs. the AR + VAN group) (Figure 3C–F).

### 3.5 Butyrylated Starch Reverses Vancomycin‐Induced Gut Microbiota Dysbiosis

**FIGURE 4:**
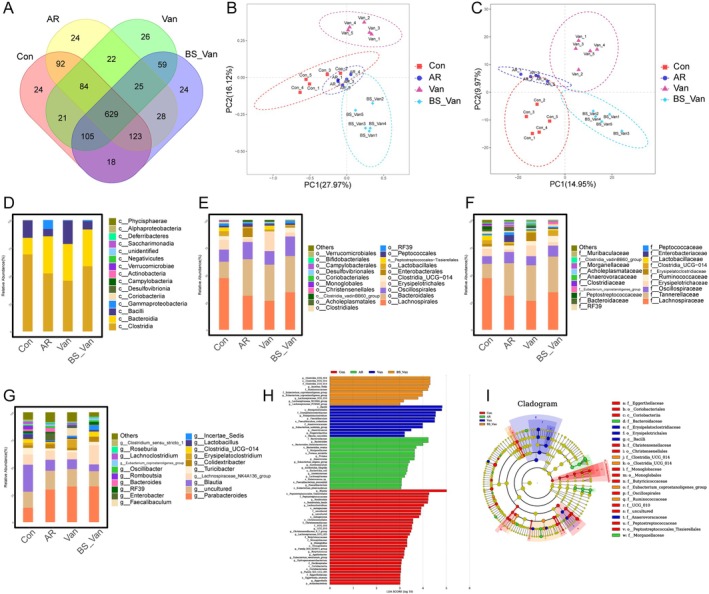
Butyrylated starch ameliorates gut microbiota dysbiosis. (A) Venn diagram showing the number of shared and unique operational taxonomic units (OTUs) among groups. (B, C) The results of the beta diversity analysis are shown by principal component analysis (PCA, B) and partial least squares‐discriminant analysis (PLS‐DA, C). (D–G) Relative abundance of bacterial taxa at the class (D), order (E), family (F), and genus (G) levels. (H, I) Linear discriminant analysis (LDA) scores from LEfSe analysis showing the most differentially abundant taxa between the AR + VAN and AR + BS + VAN groups (H) and the corresponding cladogram (I).

To explore the microbial mechanisms underlying the efficacy of BS, we performed 16S rRNA sequencing of the colonic contents. A Venn diagram revealed 629 shared OTUs across all groups (Figure 4A). Alpha diversity analysis (including Chao1, observed species, PD_whole_tree, and Shannon indices) further revealed that compared with AR supplementation, treatment with vancomycin (AR + VAN group) reduced gut microbial diversity, whereas BS supplementation (AR + BS + VAN group) partially restored these diversity indices toward the control state (Figure S2). Multivariate analyzes (PCA and PLS‐DA) confirmed that vancomycin administration induced a severe shift in the microbial community structure, which was effectively reversed by BS supplementation toward a state resembling that of the control group (Figure 4B,C). Taxonomic analysis revealed that vancomycin specifically depleted the abundance of butyrate‐producing taxa, including *Clostridia* (class level), *Lachnospirales* (order level), *Lachnospiraceae* (family level), and *Lachnospiraceae_NK4A136_group* (genus level) (Figure 4D–G). BS intervention significantly restored the abundance of these critical taxa (*p* < 0.05). LEfSe and LDA further confirmed that *Clostridia*, *Lachnospirales*, and *Lachnospiraceae* were the key discriminative features that characterized the microbial differences between the AR + VAN and AR + BS + VAN groups (Figure 4H,I).

### 3.6 Butyrylated Starch Promotes the Expansion of Splenic Regulatory T Cells

**FIGURE 5:**
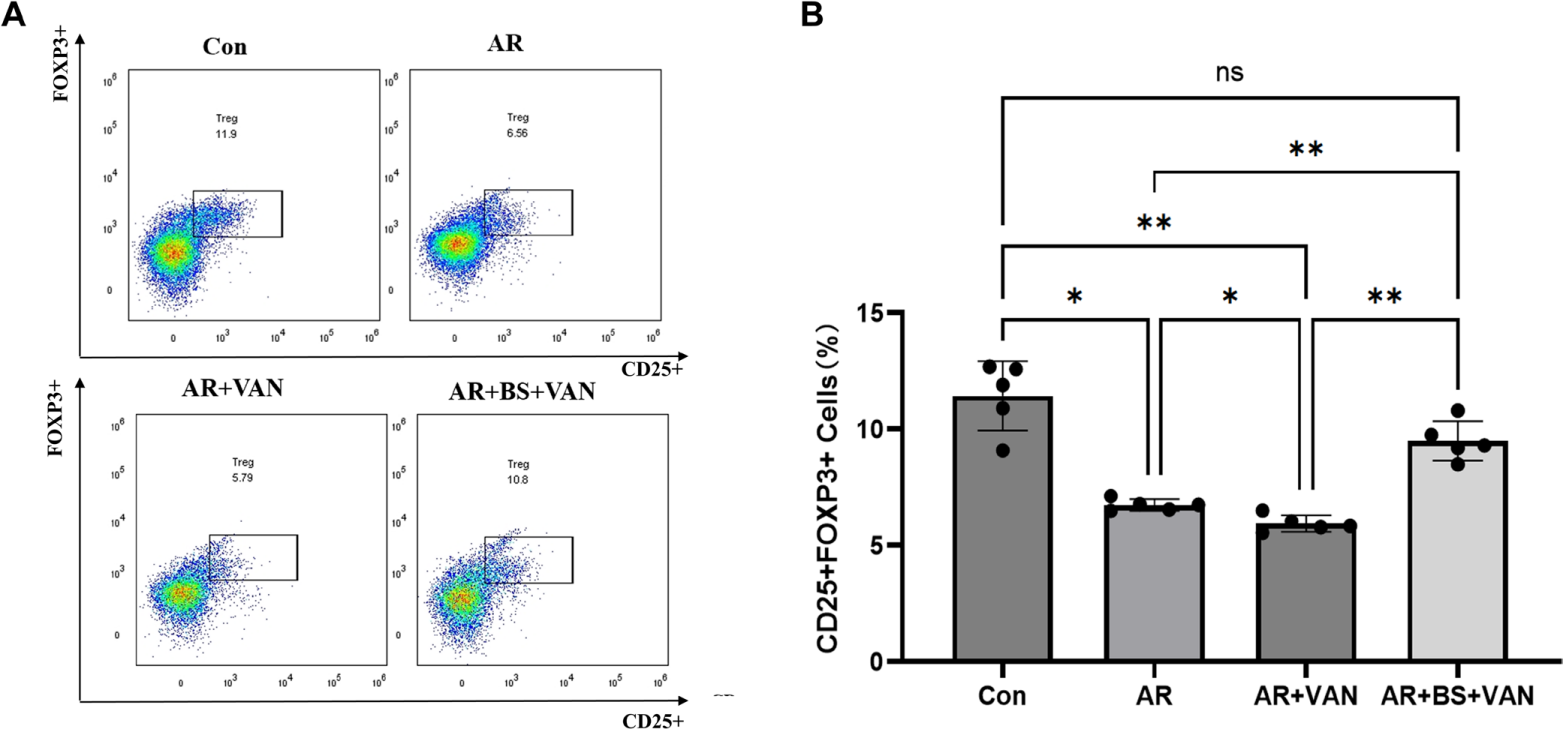
Butyrylated starch increases the proportion of splenic CD4^+^CD25^+^FOXP3^+^ regulatory T (Treg) cells. (A) Representative flow cytometry plots illustrating the gating strategy: Single cells were first selected by FSC‐A versus FSC‐H to exclude aggregates, followed by exclusion of dead cells (viability dye‐negative), gating on CD4^+^ T cells, and subsequent identification of CD4^+^CD25^+^FOXP3^+^ Treg cells. (B) Statistical summary of the percentage of Treg cells among total CD4^+^ T cells in each group. The data are presented as the means ± standard deviations (SDs) (*n* = 5). ns, not significant; *p < 0.05, **p < 0.01 (one‐way ANOVA with Tukey's post hoc test).

Finally, we examined the systemic immunomodulatory effect of BS. The proportion of splenic CD4 + CD25 + FOXP3 + regulatory T (Treg) cells was significantly lower in the AR group than in the Con group (*p* < 0.05). Vancomycin treatment further reduced the Treg cell population (AR + VAN group). Notably, BS supplementation significantly increased the proportion of splenic Treg cells in the AR + BS + VAN group compared with that in the AR + VAN group (*p* < 0.01) (Figure 5A,B).

### 3.7 Correlation Network Analysis Links Butyrate to Key Microbial Taxa and Inflammatory Mediators

**FIGURE 6:**
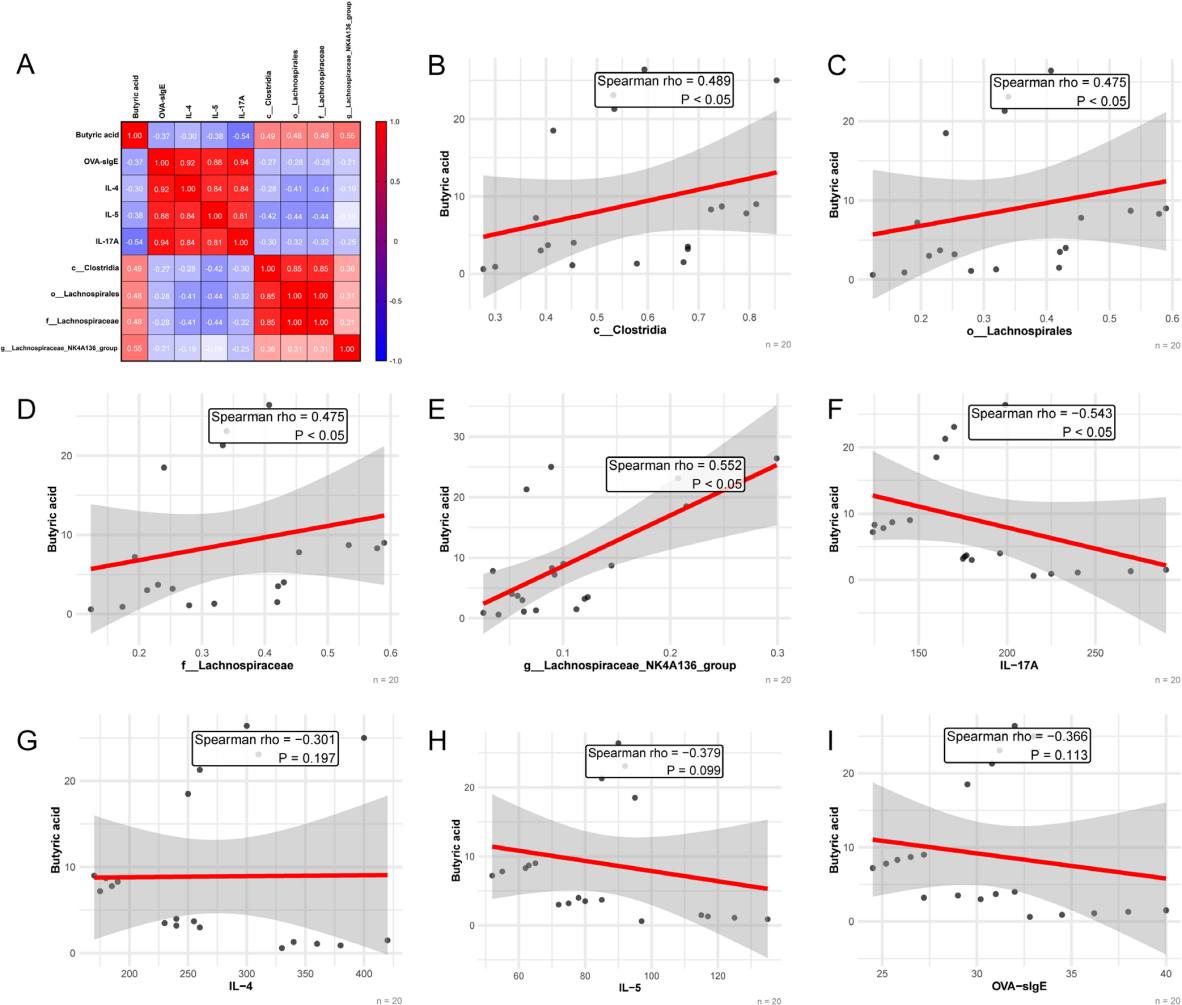
Correlation network analysis integrating butyrate, the gut microbiota, and inflammatory markers. (A) Spearman correlation heatmap of selected variables across all samples (*n* = 20). (B–I) Scatter plots illustrating the significant or trending bivariate correlations between butyric acid levels and key variables. The solid lines represent the linear fit. (B) *c__Clostridia* versus butyric acid. (C) *o__Lachnospirales* versus butyric acid. (D) *f_Lachnospiraceae* versus butyric acid. (E) *g_Lachnospiraceae_NK4A136_group* versus butyric acid. (F) IL‐17A versus butyric acid. (G) IL‐4 versus butyric acid. (H) IL‐5 versus butyric acid. (I) OVA‐sIgE versus butyric acid. Data from all the experimental groups (Con, AR, AR + VAN, and AR + BS + VAN) were pooled for this integrative correlation analysis.

To further elucidate the systemic relationships among the depleted butyrogenic microbiota, butyrate levels, and allergic inflammation, we performed a Spearman correlation analysis across all the samples. The correlation heatmap revealed a distinct network of associations (Figure 6A). Most notably, butyric acid levels were strongly positively correlated with the relative abundances of key butyrate‐producing taxa, including *c__Clostridia* (*r* = 0.489, *p* < 0.05), *o__Lachnospirales* (*r* = 0.475, *p* < 0.05), *f__Lachnospiraceae* (*r* = 0.475, *p* < 0.05), and *g__Lachnospiraceae_NK4A136_group* (*r* = 0.552, *p* < 0.05) (Figure 6B–E). Conversely, butyric acid was negatively correlated with proinflammatory cytokines. This inverse relationship was strongest and statistically significant for IL‐17A (*r* = −0.543; *p* < 0.05) (Figure 6F), whereas trends were observed for IL‐4 (*r* = −0.301; *p* = 0.197), IL‐5 (*r* = −0.379; *p* = 0.099), and OVA‐sIgE (r = −0.366; *p* = 0.113) (Figure 6G–I). These correlation analyzes provide integrative statistical evidence supporting a direct link between vancomycin‐induced depletion of specific gut bacteria, reduced colonic butyrate, and increased systemic inflammation, thereby confirming the proposed microbiota‐butyrate‐immunity axis in antibiotic‐induced AR.

## 4 Discussion

Antibiotic‐induced gut dysbiosis is being increasingly recognized to drive the exacerbation of allergic rhinitis (AR), a highly prevalent inflammatory airway disorder that remains a persistent clinical challenge with few mechanism‐targeted interventions (Brożek et al. 2017; Duong et al. 2022). In this study, we present preliminary preclinical evidence that the activity of butyrylated starch (BS), a colon‐targeted butyrate precursor, may alleviate the exacerbation of AR aggravated by vancomycin (VAN) in a murine model. Our data suggest that the protective effects of BS are mediated through coordinated modulation of the gut microbiota‐barrier‐immune axis (Lv et al. 2025): BS supplementation rescues the depletion of key butyrate‐producing taxa, including *Clostridia* and *Lachnospiraceae*; elevates colonic butyrate concentrations; repairs impaired intestinal barrier integrity via upregulation of the tight junction proteins ZO‐1, Occludin‐1, and Claudin‐3; and restores systemic immune homeostasis through promoting the expansion of splenic regulatory T cells (Tregs). These findings validate our initial hypothesis that BS mitigates antibiotic‐exacerbated AR by targeting the gut microbial and immune microenvironment and support that BS may serve as a promising novel dietary intervention to counteract the adverse effects of antibiotic exposure on allergic airway disease progression.

Our findings corroborate and extend the growing body of evidence supporting the role of the gut‐lung (gut‐nose) axis in the pathogenesis of allergic airway diseases, suggesting that gut microbiota dysbiosis acts as a potent exacerbating factor for AR (Zanvit et al. 2015; Yang et al. 2014; Kim et al. 2018; Lee et al. 2020; Ni et al. 2019). Consistent with our previous preclinical work (Chen et al. 2022), we observed that vancomycin administration induced severe disruption of the gut microbial ecology, with marked depletion of key butyrate‐producing commensal bacteria. This depletion was accompanied by a profound reduction in colonic butyrate concentrations, creating a pathological microenvironment that predisposes patients to intestinal inflammation and impaired barrier function (Régnier et al. 2021; Desai et al. 2016; Stevens et al. 2018). The subsequent increase in circulating lipopolysaccharide (LPS) levels we detected further confirms a state of endotoxemia secondary to “leaky gut,” a well‐documented driver of systemic low‐grade inflammation that can amplify pathological responses in remote mucosal sites, including the nasal mucosa (Stevens et al. 2018). While previous work has established a correlation between gut butyrate deficiency and AR susceptibility, most related studies have focused on the anti‐inflammatory effects of exogenous butyrate supplementation (Hu et al. 2018; Yang et al. 2023), with few studies addressing the core challenge of reversing antibiotic‐induced depletion of endogenous butyrate‐producing bacteria, which is the root cause of sustained butyrate insufficiency (Fishbein et al. 2023). Our study fills this gap by demonstrating that BS can target and restore these depleted butyrogenic taxa rather than simply providing transient exogenous butyrate supplementation.

The core novelty of our preliminary work lies in the first application of BS (Li et al. 2021), a well‐validated colon‐targeted butyrate precursor, to the previously uncharacterized clinical scenario of antibiotic‐exacerbated AR (Galazzo et al. 2020). A key advantage of BS over conventional butyrate supplements (such as sodium butyrate) is its ability to resist hydrolysis and absorption in the upper gastrointestinal tract, enabling targeted release of butyrate specifically in the colon, the primary site where butyrate exerts its physiological effects on the gut microbiota and mucosal barrier (Annison et al. 2003). This targeted delivery profile was critical to the effects we observed: rather than inducing a nonspecific shift in the gut microbiota, BS selectively enriched resident butyrate‐producing taxa, including *Lachnospiraceae* and *Clostridia*, thereby sustaining endogenous butyrate production rather than providing only transient exogenous supplementation.

The restoration of colonic butyrate pools by BS likely drives its therapeutic benefits through dual, interconnected mechanisms, which is consistent with the established biological functions of butyrate as an epigenetic modulator and histone deacetylase (HDAC) inhibitor (Liu et al. 2025). First, butyrate directly promotes intestinal epithelial barrier integrity by upregulating tight junction protein expression, which we confirmed via the restoration of ZO‐1, Occludin‐1, and Claudin‐3 expression in colon tissue (Horowitz et al. 2023). Second, butyrate induces the differentiation and expansion of anti‐inflammatory Tregs, as evidenced by the increased splenic Treg frequency we observed, which in turn suppresses the aberrant Th2 and Th17 inflammatory responses that drive AR pathogenesis (Wang et al. 2023). This systemic immunomodulatory effect, originating from the gut, highlights the ability of BS to rebalance allergic immune diathesis at its source rather than only mitigating local nasal symptoms.

From a clinical perspective, these findings hold notable translational potential. Current first‐line AR treatments, including nasal topical glucocorticoids and oral antihistamines, primarily alleviate local nasal symptoms by suppressing mucosal inflammation but do not address the underlying gut microbiota dysbiosis that can drive and exacerbate AR (Chen et al. 2023). As a safe, food‐grade dietary supplement, BS could act as a complementary adjunct to conventional AR therapies, targeting the systemic immune and microbial disturbances induced by antibiotic exposure to provide synergistic therapeutic benefits for patients with antibiotic‐exacerbated AR.

While our preliminary findings are promising, several important limitations must be acknowledged to contextualize the scope of this work. First, as a preclinical study conducted exclusively in a murine model, our findings have inherent translational constraints. Significant differences in gut microbiota composition, mucosal immune regulation, and AR pathophysiology exist between mice and humans (Hugenholtz and de Vos 2018); thus, well‐designed clinical trials are warranted to validate the efficacy, safety, and optimal dosing regimen of BS in human patients with AR, particularly those with a history of recent antibiotic exposure. Second, our study focused solely on vancomycin‐induced dysbiosis, which represents only one clinical scenario of antibiotic exposure (Russell et al. 2013). Given the widespread use of broad‐spectrum antibiotics in clinical practice, future studies are needed to explore whether BS exerts similar protective effects against AR exacerbation induced by other classes of antibiotics or by combined antibiotic regimens.

In terms of mechanism, our study revealed a causal role for the microbiota‐barrier‐immune axis in mediating the protective effects of BS but did not fully elucidate the specific molecular pathways through which gut‐derived butyrate modulates nasal mucosal immunity. Butyrate is known to exert its biological effects via multiple pathways, including the activation of G protein‐coupled receptors (GPR43, GPR109a, and GPR41) and HDAC inhibition (Marchix et al. 2018; Singh et al. 2014; Chang et al. 2014); future in vitro and in vivo studies are needed to define the specific molecular targets and cell subsets through which butyrate regulates the gut‐nose axis in the context of AR (Yu et al. 2025). Additionally, further investigations are needed to characterize the effects of BS on nonbutyrate‐producing microbial taxa, its long‐term impact on gut microbiota stability, and whether interindividual differences in the human gut microbiome may lead to variable responses to BS supplementation.

In conclusion, our preliminary study systematically characterized a novel dietary intervention strategy for antibiotic‐exacerbated AR. We move beyond correlative associations to define a causal pathological cascade: antibiotic‐induced gut dysbiosis drives the depletion of butyrate‐producing commensals, which in turn leads to colonic butyrate insufficiency, intestinal barrier disruption, systemic immune dysregulation, and ultimately exacerbated AR. BS intervention reverses this pathological cascade at its source by restoring the gut butyrate‐producing microbial community and coordinating the repair of the microbiota‐barrier‐immune axis. By leveraging the host's gut microbiota to restore a key metabolic and immunomodulatory mediator, BS offers a safe, practical, and mechanism‐based dietary approach to manage allergic inflammation, with potential applicability to other inflammatory conditions linked to antibiotic‐induced gut dysbiosis (Ney et al. 2023).

## 5 Conclusion

In conclusion, this study systematically validated our initial hypothesis that butyrylated starch (BS) mitigates vancomycin‐exacerbated allergic rhinitis (AR) via coordinated modulation of the gut microbiota‐barrier‐immune axis. We demonstrate that vancomycin‐induced gut dysbiosis drives AR aggravation by depleting butyrate‐producing commensal bacteria, reducing colonic butyrate levels, disrupting intestinal barrier integrity, and amplifying systemic allergic inflammation. As a colon‐targeted butyrate precursor, BS reverses this pathological cascade at its source: it rescues the depletion of key butyrate‐producing taxa, including *Clostridia* and *Lachnospiraceae*; restores intestinal barrier function through upregulation of tight junction proteins; and rebalances immune homeostasis by promoting Treg cell expansion and inhibiting Th2/Th17‐mediated inflammatory responses. Our preliminary findings establish BS as a novel, safe, and microbiota‐targeted dietary intervention with promising translational potential for the management of antibiotic‐exacerbated AR. Future clinical trials are needed to validate the efficacy, long‐term safety, and optimal dosing of BS in human AR patients, especially those with antibiotic‐associated gut dysbiosis, to advance this preclinical insight into a clinically available nonpharmacological therapeutic option for allergic airway disease.

## Author Contributions

**Xing Lin:** methodology, data curation. **Yuxi Lin:** writing – original draft, writing – review and editing, validation, methodology, data curation, conceptualization. **Jingmin Chen:** writing – original draft. **Yang Liu:** methodology, investigation, data curation. **Qingqing Xu:** methodology, conceptualization, funding acquisition. **Yuanteng Xu:** writing – review and editing, project administration, funding acquisition. **Siyi Tang:** validation, formal analysis, data curation.

## Funding

This was funded by the National Natural Science Foundation of China (No. 82271143); the Medical Innovation Project of Fujian Provincial Health Commission (No. 2023CXA024); the Joint Funds for the Innovation of Science and Technology, Fujian Province (No. 2023Y9053); the Startup Fund for Scientific Research of Fujian Medical University (No. 2022QH1207); and the Startup Fund for Scientific Research of Fujian Medical University (No. 2022QH1798).

## Conflicts of Interest

The authors declare no conflicts of interest.

## Supporting information


**Figure S1:** Cumulative symptom scores (sum of graded sneezing and scratching scores) in each group. The data are presented as the means ± SDs (*n* = 5). *p* < 0.01 (one‐way ANOVA with Tukey's post hoc test).
**Figure S2:** Alpha diversity analysis of the gut microbiota among the different groups.
**Table S1:** Animal feed composition list.
**Table S2:** Mouse AR symptom score table.

## Data Availability

The data that support the findings of this study are available from the corresponding author upon reasonable request.
