# Supplementary material for: Butyrylated Starch Attenuates Antibiotic‐Exacerbated Allergic Rhinitis by Restoring the Gut Microbiota and Barrier Function in Mice
Source: Food Sci Nutr. 2026 Jun 7;14(6):e72000. doi: 10.1002/fsn3.72000 (PMC13243702; doi:10.1002/fsn3.72000)
Supplement: Supplementary file 1 — Figure S1: Cumulative symptom scores (sum of graded sneezing and scratching scores) in each group. The data are presented as the means ± SDs (n = 5). p < 0.01 (one‐way ANOVA with Tukey's post hoc test). Figure S2: Alpha diversity analysis of the gut microbiota among the different groups. Table S1: Animal feed composition list. Table S2: Mouse AR symptom score table. [file FSN3-14-e72000-s001.docx]

**Materials and methods**

**Preparation of the Butyrate Starch**

Ordinary corn starch was purchased from Shandong Juneng Golden Corn Co., Ltd.; butyric anhydride (analytically pure) was purchased from Shanghai Aladdin Biochemical Technology Co., Ltd.; and other chemical reagents (analytically pure) were purchased from Guoyao Group Chemical Reagent Co., Ltd.

A certain quantity of ordinary corn starch was weighed, transferred to a four-necked flask, 40% (m/v) starch milk was prepared. The four-necked flask was placed in a water bath, the mixture was continuously stirred, and the reaction temperature was adjusted to 40°C. Butyric anhydride (controlled to drip within 2 h, the amount of anhydride was 40% (m/m) on a starch dry basis) was slowly added, and 0.75 mol/L NaOH solution was added at the same time to maintain the pH value of the reaction system at 8.0-8.5. After the addition of anhydride, the reaction was continued for 2 h, and the pH of the reaction system was maintained between 8.0 and 8.5. After the reaction was complete, the pH was adjusted to neutral with 1 mol/L HCl to terminate the esterification reaction. The samples were washed with distilled water multiple times, centrifuged to collect starch, dried overnight at 40°C, crushed, and passed through a 100 mesh sieve to obtain butyrate starch products, which were then sealed and ready for use. For the determination of the butyryl content, GB 29923-2013 Determination Method of Acetyl Group Content in Acetate Starch was used, and the degree of substitution (DS) was calculated according to the following formula. The measured DS of the prepared sample was 0.237. The DS is calculated via the following formula:

$$\text{DS = }\frac{\text{162 × w}}{\text{7100-70 × w}}$$

**Supplementary Table 1**. Animal feed composition list.

| **Formula (per 1000 g of feed)** | **Normal starch feed** | | **Butylated starch feed** | |
| --- | --- | --- | --- | --- |
|  | Gram | Kcal | Gram | Kcal |
| Normal Corn starch | 150 | 600 | - | - |
| Butylated starch | - | - | 150 | 420 |
| Casein | 200 | 800 | 200 | 800 |
| L-Cystine | 3 | 12 | 3 | 12 |
| Corn starch | 249.486 | 998 | 239.486 | 958 |
| Maltodextrin | 130 | 520 | 140 | 560 |
| Sucrose | 100 | 400 | 100 | 400 |
| Cellulose | 50 | 0 | 50 | 0 |
| Soybean Oil | 70 | 630 | 70 | 630 |

**Supplementary Table 2.** Mouse AR symptom score table.

| symptom | mild（1 point） | | moderate（2point） | severe（3point） |
| --- | --- | --- | --- | --- |
| Rubs | | 1-5 | 6-15 | >15 |
| Sneezes | | 1～3 | 4～10 | >10 |
| runny nose | | Flowing to the front nostrils | Beyond the front nostrils | Flowing all over the face |

**Supplementary Figure 1**


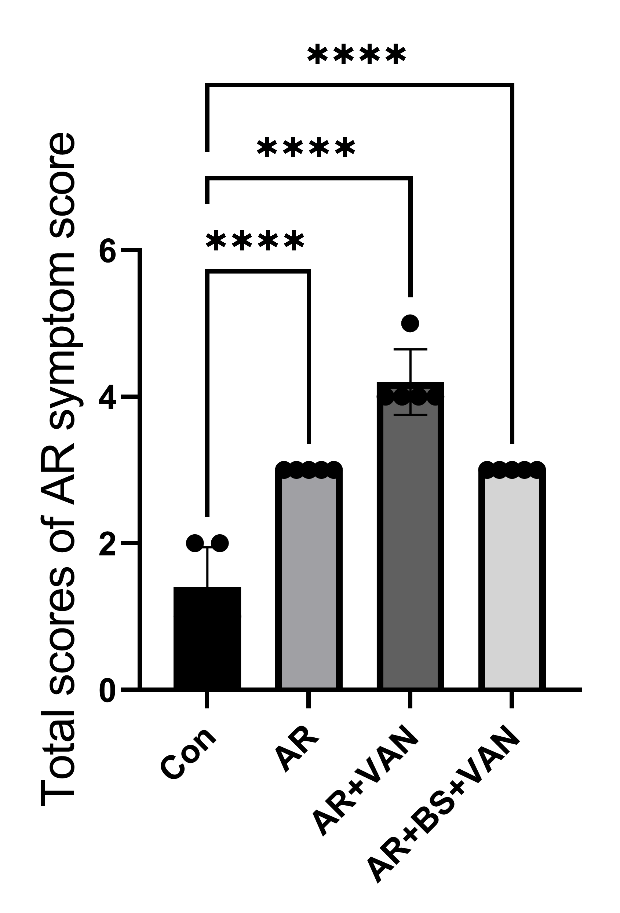


**Supplementary Figure 1.** **Cumulative symptom scores (sum of graded sneezing and scratching scores) in each group.** The data are presented as the means ± SDs (n=5). *p* < 0.01 (one-way ANOVA with Tukey's post hoc test).

**Supplementary Figure 2**

**
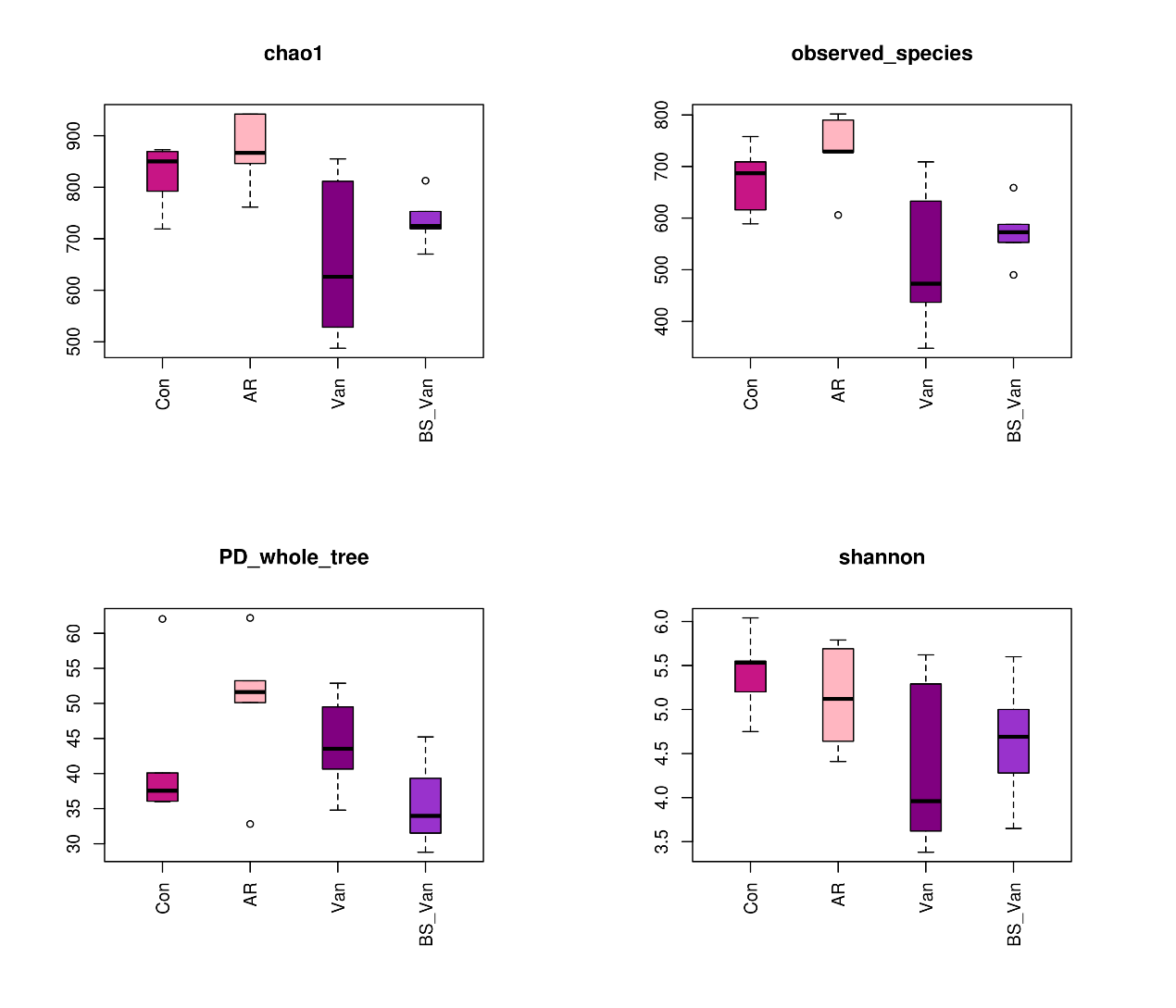
**

**Supplementary Figure 2. Alpha diversity analysis of the gut microbiota among the different groups.**

(A) Chao1 index (reflecting microbial species richness);

(B) Observed_species index (reflects detectable microbial richness via observed operational taxonomic units, OTUs);

(C) PD_whole_tree index (reflecting microbial phylogenetic diversity by integrating interspecies evolutionary relationships);

(D) Shannon index (reflecting comprehensive microbial diversity, considering species richness and evenness).

The data are presented as the means ± standard deviations (SDs) (n=5). ns, not significant; **p* < 0.05, ***p* < 0.01 (one-way ANOVA with Tukey's post hoc test).
